# Clinical and Genetic Functional Validation of a Novel *AP1S1* Mutation Causing MEDNIK Syndrome

**DOI:** 10.1155/ijog/4385128

**Published:** 2025-08-25

**Authors:** Lifen Duan, Ru Shen, Guoyan Yin, Ruixi Tao, Yi Zhang, Wei Yu, Lishimeng Bao, Weitao Ye, Runxiu Yin, Xin Tian

**Affiliations:** ^1^Epilepsy Center, The Affiliated Children's Hospital of Kunming Medical University, Kunming Medical University, Kunming, China; ^2^Epilepsy Center, Kunming Children's Hospital, Kunming, China; ^3^Division of Laboratory, Kunming Maternity and Child Care Hospital, Kunming, China; ^4^Department of Pediatrics, Kunming Medical University, Kunming, China; ^5^Department of Hematology, The Affiliated Children's Hospital of Kunming Medical University, Kunming Medical University, Kunming, China; ^6^Department of Hematology, Kunming Children's Hospital, Kunming, China

**Keywords:** *AP1S1*, copper metabolism, MEDNIK syndrome, mutation

## Abstract

**Background:** MEDNIK syndrome is a rare copper metabolism disorder caused by *AP1S1* variants. Herein, we report the clinical and genetic characteristics of MEDNIK syndrome in two siblings.

**Methods:** The clinical treatment process for MEDNIK syndrome and over 4 years of follow-up data were analysed in two siblings. Microscopic observations of the patients' hair were conducted. Gene sequencing, three-dimensional structural reconstruction of protein sequences, and in vitro mRNA splicing experiments were performed.

**Results:** The proband and his sister exhibited developmental delays, seizures, yellow hair, sparse teeth and a high forehead. Furthermore, the sister initially presented with intractable diarrhoea and severe pneumonia. Both siblings showed varying degrees of developmental delays during follow-up, and the proband also showed symptoms of attention deficit hyperactivity disorder. The microscopic hair examination revealed a deficiency in intermediate pigment, a pale colour and an intermittent or absent medulla. Genetic sequencing revealed a homozygous *AP1S1* mutation at the splicing site (NM_001283.3): c.430-1G>A. The in vitro mRNA splicing experiments confirmed a single base-pair deletion in the fifth exon of the mRNA sequence of the mutated plasmid, resulting in a frameshift mutation (p.Glu144ArgfsTer83). The mutation was inherited from both parents and classified as pathogenic according to the American College of Medical Genetics and Genomics guidelines, based on clinical features and family analysis.

**Conclusion:** Both children with MEDNIK syndrome exhibited heterogeneous clinical phenotypes. Sparse teeth may be a previously unnoticed feature of MEDNIK syndrome. The pathogenic c.430-1G>A homozygous variant enriches the mutation spectrum of *AP1S1*. This mutation causes a frameshift mutation in the protein, altering the protein structure and affecting protein function.

## 1. Introduction

MEDNIK syndrome, first discovered by Saba et al. in 2005, is a rare autosomal recessive disorder of copper metabolism, characterised by mental retardation, enteropathy, deafness, peripheral neuropathy, ichthyosis and keratoderma [1]. Consequently, Montpetit et al. coined the term MEDNIK syndrome, an acronym of its clinical features, in 2008 [2]. MEDNIK syndrome is a multisystem disorder caused by a mutation in *AP1S1*, leading to copper absorption or transport dysfunction. Mutations in *AP1S1* can result in abnormal trafficking of copper transporters mediated by adaptor protein complex 1 (AP-1), abnormal intracellular transport of copper pumps, secondary deficiency of copper-dependent enzymes and copper transport disorders. Patients with MEDNIK syndrome exhibit clinical and biochemical features of other copper metabolism disorders such as Menkes syndrome and Wilson's disease [3]. Owing to the absence of distinct phenotypic features, diagnosing MEDNIK syndrome is a challenge.

To date, only 14 cases of MEDNIK syndrome have been reported worldwide. In this study, we analysed the data of two affected siblings. Herein, we summarise the clinical and genetic characteristics of MEDNIK syndrome in the two siblings, including 4-year follow-up data. To our knowledge, this is the first report of siblings with MEDNIK syndrome in China.

## 2. Methods

### 2.1. Patients and Ethical Considerations

Clinical data of two patients with MEDNIK syndrome treated at Kunming Children's Hospital were analysed. This study was approved by the Ethics Committee of Kunming Children's Hospital (2021-03-055-K01), and written informed consent was obtained from the parents of the patients.

### 2.2. Microscopic Examination of Hair Colour and Textural Changes

A small section of hair, close to the root, was cut for sample fixation and placed on a microscope slide. A standard microscope solution was applied, and the sample was covered with another slide. External features of the hair, such as colour, shape and size, were observed. Concurrently, the internal structures of the hair, including the hair scales, cortical layers and medulla, were observed.

### 2.3. Whole Exome Sequencing

Peripheral blood samples (2 mL) were collected from the patients, their parents and the other sibling. Genomic DNA was extracted from the peripheral blood of the progenitors and their family members. After fragmentation, splicing, amplification and purification, a DNA library was prepared using the hybridisation capture method. A high-throughput sequencing platform (Illumina NovaSeq 6000; Illumina, United States) was used to identify the exon and intron regions of the entire human exome (20 bp). The sequencing data were compared with the human genome reference sequence hg19 (GRCh37), and coverage of the target region and sequencing quality were evaluated. In this assessment, we analysed copy number variants (CNVs) of large segments (two or more consecutive exons) of the listed genes, and the reported CNVs were validated using orthogonal tests (quantitative polymerase chain reaction [PCR] or multiplex ligation–dependent probe amplification). Public databases (ESP6500, 1000 Genomes, ExAC and GnomAD) were used to filter out variants at frequencies higher than 0.001. Splice site prediction software (Human Splicing Finder [HSF] system and dbscSNV Version 1.1 software) was used to predict pathogenic single nucleotide variants. After sequencing, genetic analysis was performed according to the ACMG guidelines for pathogenicity assessment, combined with the analysis of the clinical data of the children. PCR amplification was performed to obtain samples for Sanger sequencing to verify the mutations and genetic patterns identified through WES. The sequencing results were compared and verified using MutationSurveyor software.

### 2.4. In Vitro mRNA Splicing (Minigene)

To investigate the splicing impact of the AP1S1 c.430-1G>A variant identified by WES, we performed a minigene splicing assay using HEK293T cells. Wild-type and mutant minigene constructs encompassing Exons 4 and 5 of AP1S1 (NM_001283.3) were generated. The wild-type plasmid was constructed by amplifying genomic DNA through seamless cloning, while the mutant was introduced using site-directed mutagenesis based on the wild-type backbone. Recombinant plasmids were verified by Sanger sequencing. Validated minigenes were transfected into HEK293T cells. After 48 h, total RNA was extracted using the TRIzol method and quantified. Reverse transcription was performed using 1 *μ*g RNA per reaction, followed by RT-PCR to amplify splicing products. PCR amplicons were analysed via agarose gel electrophoresis and subjected to Sanger sequencing. Two independent clones were sequenced per construct to confirm reproducibility. Given the low expression of AP1S1 in peripheral blood and ethical limitations in accessing patient tissue, we used an in vitro splicing system. Although indirect, this minigene assay offers a reliable approach to assess the effect of splice-site mutations under controlled conditions and is widely used for evaluating transcript-level changes.

#### 2.4.1. Note on RNA Source and Validation Strategy

Given the limited expression level of *AP1S1* in peripheral blood and the ethical constraints associated with obtaining patient-derived tissue samples, we did not perform RT-PCR directly on patient RNA samples. Instead, we employed an in vitro minigene assay using HEK293T cells, which allowed for controlled analysis of the splicing consequences of the *AP1S1* c.430-1G>A variant. This system enabled us to mimic the splicing environment and evaluate transcript-level changes between the wild-type and mutant constructs. Although not a direct patient RNA validation assay, the minigene assay remains a widely accepted and informative approach for assessing splicing defects caused by intronic or splice-site mutations.

### 2.5. Protein Structure Modelling and Visualisation

The three-dimensional structures of the wild-type and mutant *AP1S1* proteins were predicted using the SWISS-MODEL web server (https://swissmodel.expasy.org) based on the amino acid sequences translated from the canonical transcript (NM_001283.3) and mutant frameshift variant (p.Glu144ArgfsTer83). Homology modelling was performed using the NPR-C crystal structure (PDB ID: 1JDP) as the template, selected based on the highest sequence identity. The predicted models were aligned and visualised using PyMOL (Version 2.5.4, Schrödinger, LLC). Structural differences between the wild-type and mutant proteins were highlighted to illustrate the conformational changes caused by the splice-induced frameshift mutation.

### 2.6. Follow-Up Assessment

We followed up the siblings for 4 years, regularly performed laboratory tests and conducted detailed developmental assessments.

## 3. Results

### 3.1. Clinical Information

**Figure 1:**
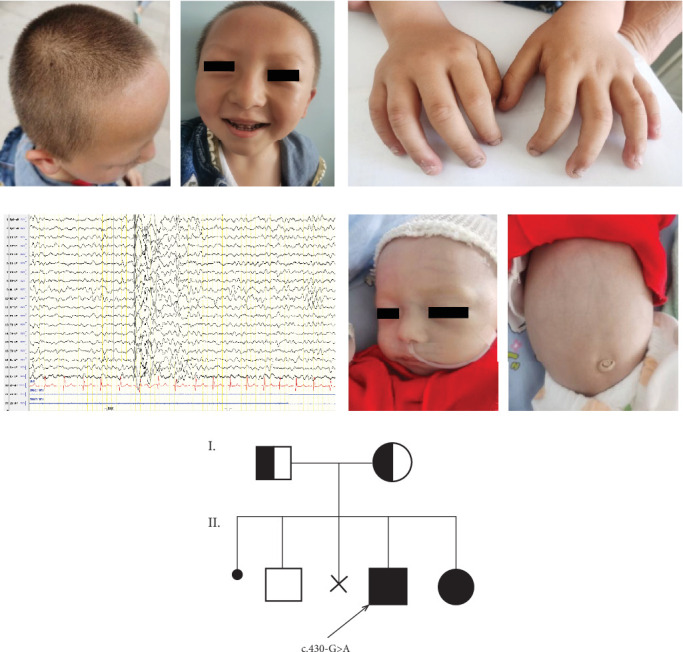
Facial features, skin and nail changes, VEEG and genetic family pedigree. (a) Facial features of Case 1: high forehead, pale eyebrows, wide inter-eye distance and sparse teeth. (b) Local nail defects and surrounding skin desquamation in Case 1. (c) The VEEG showed widespread 1.5–2.5 Hz spike-slow waves during sleep, with prominent activity in the anterior head in Case 1. (d) The facial features of Case 2: white skin, pale eyebrows and abdominal distension. (e) Family pedigree.

Herein, we report two siblings newly diagnosed with MEDNIK syndrome. The proband (a boy aged 5 year and 3 months; Case 1) has one healthy elder brother and one affected younger sister (Case 2) (Figure 1e). The proband presented with primary symptoms including intellectual and language developmental disorders, seizures, pale skin and hair pigmentation. At 2 months of age, the child experienced convulsive seizures. The child received levetiracetam (30 mg/kg/day) for the treatment of epileptic seizures. He was treated with lysine hydrochloride and zinc gluconate granules (zinc content, 5 mg bid) for 4 months. The proband had a high forehead, fair skin, yellow hair, very light-coloured eyebrows, widened interocular distance, sparse teeth and pigmentation abnormalities. The distal parts of his nails were partially missing and uneven, with the surrounding skin appearing slightly rough and flaky (Figure 1a,b). Hyperkeratosis, increased skin hardness, and enlarged pores were observed on the skin of the wrist and back. No visual or hearing abnormalities were noticed. An auxiliary examination at 3 months of age revealed a ceruloplasmin (CER) level of 0.11 g/L, and the alkaline phosphatase (ALP) was 1085 U/L. Iron, zinc and lead levels; thyroid and adrenal functions and cerebrospinal fluid (CSF) routine biochemistry results were normal. Brain magnetic resonance imaging (MRI) findings and video electroencephalogram (VEEG) were also normal. Urine organic acid gas phase mass spectrometry and blood tandem mass spectrometry results were normal. The urinary copper level was 12.2 *μ*g/24 h, and the Kayser–Fleischer (K–F) ring test was negative. At 5 years and 3 months of age, VEEG showed widespread 1.5–2.5 Hz spike-slow waves during sleep, with prominent activity in the anterior head (Figure 1c). A mental scale assessment for children aged 0–6 years (63.5 months) revealed developmental delays, the social adaptability crude score was 26 and the standard score was moderately abnormal. Pure-tone audiometry indicated normal hearing.

Case 2 involves a 3-month-old girl, the younger sister of the proband, delivered via a caesarean section at 36 weeks + 5 days of gestation. She developed cyanosis 40 min after birth and was diagnosed with severe pneumonia. Despite anti-infection treatment, the patient remained supplemental oxygen-dependent and presented with recurrent abdominal distension and intractable diarrhoea, experiencing more than 10 watery stool events per day. At 59 days of age, the patient experienced seven convulsions in a single day, each lasting approximately 1 min. Following treatment with levetiracetam (30 mg/kg/day), the symptoms were controlled. Lysamine–glucozinc particles (zinc content 2.5 mg/d) were administered, reducing the frequency of diarrhoea to 2–4 times per day after 2 months of treatment. Stool moisture level decreased significantly, with occasional abdominal distension and slow deoxygenation, and there were no further convulsive seizures. The patient presented with a generalised poor health condition and response, inability to raise the head, fair skin and hair, faintly coloured eyebrows and a thin subcutaneous fat layer (Figure 1d). Auxiliary tests revealed CER level of 0.06 g/L and ALP level of 1709 U/L. Thyroid function, blood gas analysis, blood ammonia examination and CSF routine biochemistry results were normal. Urine organic acid gas phase mass spectrometry and blood tandem mass spectrometry results were normal. VEEG showed delayed maturation of brain electrical activity in combination with her gestational age. Liver ultrasonography and brain MRI findings were normal.

### 3.2. Microscopic Changes in Hair Colour and Texture

**Figure 2:**
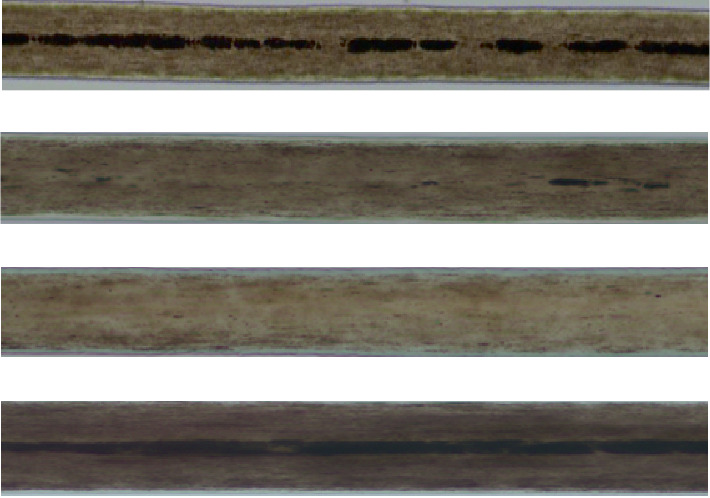
Microscopic examination of the hair morphology in the two patients with MEDNIK syndrome and a normal child (×200 magnification). (a, b) The medullary layer displayed an intermittent myelin sheath, and some areas of the myelin sheath had disappeared in the proband (Case 1). (c) The hair of the proband's sister was characterised by less pigmentation, lighter colouration and loss of the myelin sheath. (d) The hair of normal children is characterised by an even distribution of both pigment and medulla.

Microscopic hair examination of the proband revealed structural alterations. The cuticle of the outer epidermis exhibited a scaly arrangement, although the layer appeared to be relatively homogeneous and indistinct. The middle layer demonstrated reduced pigmentation and lighter colouration. The medullary layer displayed an intermittent myelin sheath, and some areas of the myelin sheath had disappeared (Figure 2a,b). The hair of the proband's sister exhibited structural changes under the microscope. The outer epidermis was homogeneous and keratinised with poor gradation, whereas the middle layer had less pigmentation and lighter colouration. The medullary layer was characterised by the loss of the myelin sheath (Figure 2c). The hair of neither child showed any of the changes typical of Menkes syndrome, such as curly texture; uneven thickness or hollow, twisted or broken strands.

### 3.3. Genetic Sequencing Results

**Figure 3:**
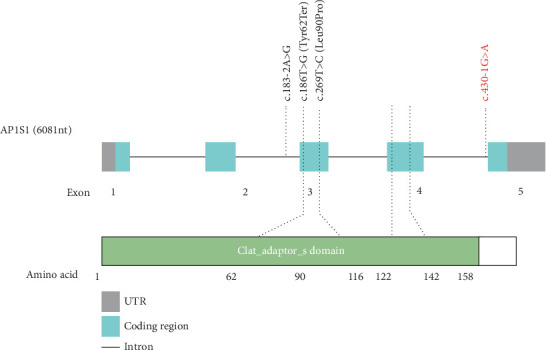
Gene functional domains (diagram of all variants, with red font indicating the variants identified in this study).

**Figure 4:**
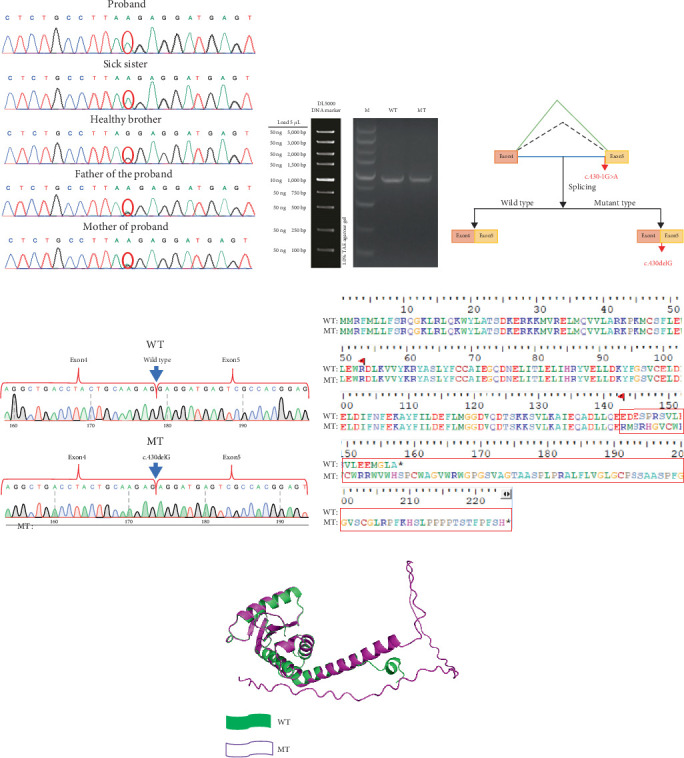
Sanger sequencing, RT-PCR agarose gel electrophoresis image, minigene and three-dimensional structure of the protein. (a) Sanger sequencing peak shows that the mutant plasmid mRNA was c.430delG. (b) RT-PCR agarose gel electrophoresis image. (c) Wild-type plasmid containing complete exons and mutant plasmid 5 exon missing 1 bp of sequence. (d) Wild-type and mutant protein sequences. (e) Three-dimensional structure of the protein shows that the structure of the mutant had apparently changed. Green represents the wild-type, and purple represents the mutant type.

Using Trio-WES, a homozygous mutation (c.430-1G>A) was identified in the splicing site of *AP1S1* in the two siblings. Their parents were also tested and found to have a heterozygous mutation at this site, indicating autosomal recessive inheritance. According to the ACMG guidelines, the c.430-1G>A variant is classified as pathogenic; its gene distribution is illustrated in Figure 3. This variant has not been previously reported in the HGMD or CLINVAR databases, and the older brother of the patients did not carry this mutation (Figure 4). The c.430-1G>A mutation substitutes guanine with adenine, resulting in an amino acid change at splice-3 (3⁣′-end splicing mutation), which is a splice-site mutation that has a substantial effect on gene function. According to our speculation and dbscSNV 1.1 and HSF website software prediction, the mutation affects protein splicing (AdaScore and RFScore > 0.6 influence splicing) (Table 1). The locus had an AdaScore of 0.999 and RFScore of 0.938, both of which affect the function of this splice site and have a significant effect on protein function. This may affect the overall conformation and activity of the protein. *AP1S1* has been reported to be associated with MEDNIK syndrome progression.

### 3.4. In Vitro mRNA Splicing Experiment (Minigene)

Through a comprehensive comparison of the expression products of the wild-type and mutant minigene vectors in cells, we observed the specific effects of *AP1S1* variation on the mRNA splicing process. In the PCR amplification experiment, the expected sequence length for the control group was 850 bp. The experimental results showed that the mRNA sequence transcribed by the wild-type plasmid, including Exons 4 and 5, was as expected. In contrast, the mutant plasmid showed a unique mRNA transcription pattern; there was a 1-bp deletion in Exon 5, which was specifically represented in the mRNA as NM_001283.3:c.430delG (Figure 4b,c). The mRNA sequence was analysed via Snap gene translation, and the variant protein was identified as p.Glu144ArgfsTer83. The amino acid glutamic acid (Glu), which was originally located at the 144th position in the protein sequence, was replaced with arginine (Arg), and this replacement triggered a frameshift mutation. The original stop codon was inactivated, and a new stop codon (Ter) was produced at the original splice-3 (3⁣′-terminal splicing mutation), enabling protein translation to continue (Figure 4d). This finding demonstrates how mutations not only alter a single amino acid but may also ensure extended protein synthesis, resulting in a protein that is elongated and with potential loss of its original function. The experimental results were highly consistent with the predicted outcomes of the protein cleavage site based on AdaScore (0.999) and RFScore (0.938), further confirming that the mutation affects the functionality of the cleavage site. These results indicate that the c.430-1G>A variant abolishes the canonical 3⁣′ splice acceptor site at the Intron 4/Exon 5 junction. A cryptic acceptor site located 1 bp downstream is likely activated, resulting in the deletion of the first nucleotide of Exon 5 (c.430delG). This splicing event leads to a frameshift and introduces a novel premature termination codon. The outcome is consistent with the splicing predictions of dbscSNV and HSF. Both sequenced mutant clones yielded the same c.430delG deletion, confirming the reproducibility of the splicing event.

### 3.5. Three-Dimensional Structure Alignment

Owing to the deletion of the G base at position 430, a frameshift mutation occurred, resulting in a protein sequence with a higher number of amino acids than that in the mutant-type (Figure 4e). Three-dimensional structure alignment showed that there were still many overlapping regions between the wild-type and mutant-type sequences, indicating that the main structure was not completely disrupted. However, the mutated protein had more loops, which were more curved and dispersed, suggesting that the structural stability of the protein may have decreased and its function may have been altered after the mutation.

### 3.6. Treatment and Follow-Up

#### 3.6.1. Case 1

**Figure 5:**
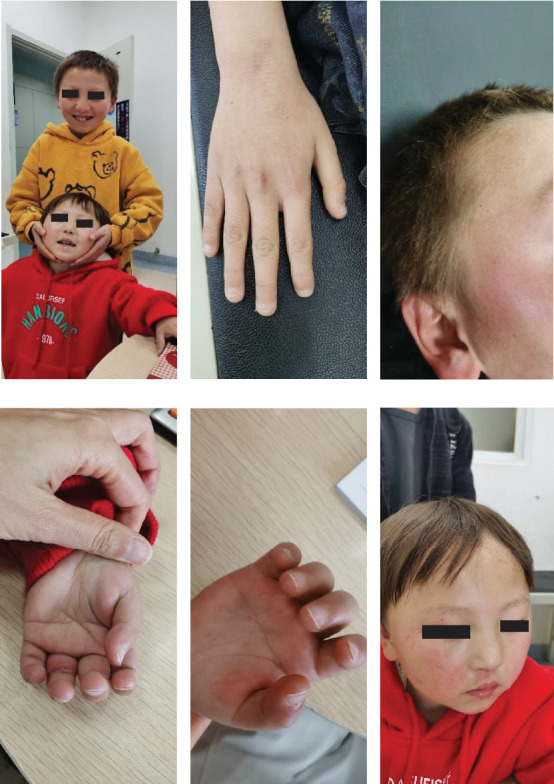
Facial features and hair characteristics of the two siblings during a follow-up visit. (a) The siblings have yellow hair, a high forehead, pale eyebrows and sparse teeth. (b) Local nail defect and surrounding skin desquamation in the elder brother. (c) Yellow hair in the proband. (d) Peeling nails in the proband's sister. (e) The simian crease in the proband's sister. (f) Yellow hair, a high forehead and pale eyebrows in the proband's sister.

The proband was administered levetiracetam oral solution for 4 years, and there were no epileptic seizures. The medication was discontinued at the age of 6 years. He is 9 years old and exhibiting global developmental delays. Although the patient is capable of jogging at a slow pace, his motor coordination remains impaired. He has yellow hair, fair skin, sparse and protruding teeth and partial nail loss on both hands, with desquamation around the nails (Figures 5a, 5b, and 5c). Laboratory tests showed a CER level of 0.02 g/L. The VEEG findings were also normal. We conducted a comprehensive developmental assessment of the child and found that he has severe intellectual disability and attention deficit hyperactivity disorder (ADHD) (Table 2).

#### 3.6.2. Case 2

The sister of the proband had no epileptic seizures at 7 months of age when on levetiracetam, and the parents stopped the treatment. However, she had a recurrence of seizures at 2 years and 2 months of age and ultimately resumed taking levetiracetam. However, the epileptic seizures persisted. She was then treated with an oral solution of sodium valproate (21 mg/kg/day) in combination with levetiracetam for 2.5 years without any epileptic seizure events. Currently, the child is 4 years and 8 months old, with delayed language and cognitive development but without epileptic seizures. She has fair skin, blonde hair, a high forehead, light-coloured eyebrows, sparse teeth, a simian crease and a positive bilateral Babinski sign (Figures 5d, 5e, and 5f). Liver function results, phenylalanine level, and ALP level were normal. The CER level was 0.34 g/L. The EEG showed increased *θ* activity in the background. Her developmental scale assessment indicated moderate developmental delays (Table 2).

## 4. Discussion

MEDNIK syndrome is a rare genetic disease associated with copper metabolism. Copper is essential for human growth and development and is a key factor in biological processes such as cell respiration, neurotransmitter synthesis, skin pigmentation, iron metabolism and oxide disproportionation [4, 5]. The stability of copper metabolism is related to food intake; gastrointestinal absorption; blood transport; hepatobiliary excretion and intracellular copper uptake, distribution and storage [6]. The most common hereditary copper metabolic diseases are Wilson's and Menkes syndrome, with rare reports of MEDNIK syndrome.

Homozygous mutations in the splice site region were found at the c.430-1G>A locus of *AP1S1* in our patients' family, which led to changes in the amino acid sequence. Analysis using splicing function prediction software indicated that this mutation affected the function of the splicing site. The c.430-1G>A variant disrupts the highly conserved AG dinucleotide of the canonical 3⁣′ splice site. As predicted using bioinformatics tools and confirmed using the minigene experiment, this loss of the natural splice site causes recognition of a cryptic splice acceptor immediately downstream. This results in an aberrant mRNA with a 1-bp deletion at the start of Exon 5, a frameshift in translation and the formation of a premature stop codon. This mechanism highlights the critical role of acceptor site integrity in precise exon definition.

Our in vitro mRNA splicing experiments verified that the c.430-1G>A variant causes a frameshift mutation in the protein, significantly altering the protein structure and considerably affecting protein function. This mutation was evaluated as pathogenic according to the ACMG guidelines, combined with the clinical characteristics of the patients. Searches on the HGMD and CLINVAR websites revealed no previous reports of this mutation. Mutations in *AP1S1* can lead to MEDNIK syndrome. The two patients in this study were diagnosed with MEDNIK syndrome based on WES results and in vitro mRNA splicing experiments, in conjunction with the clinical characteristics.

*AP1S1* is located in the 7q22.1 region of the human chromosome and encodes the *σ*1 subunit of AP-1 [7]. AP-1 connects vesicles to clathrin and plays a role in protein transport during endocytosis. The Golgi apparatus regulates clathrin-coated vesicle assembly and vesicle transport in eukaryotic cell organelles. *AP1S1* mutations result in AP-1-mediated abnormal trafficking of copper transporters [3], resulting in abnormal intracellular transport of the copper pumps ATP7A and ATP8A, secondary deficiency of copper-dependent enzymes and impaired copper transport. The resulting hypocopper condition, hypoceruloplasminemia and accumulation of copper in the liver contribute to the clinical and biochemical phenotypes of MEDNIK syndrome, which exhibit similarities to Menkes syndrome and Wilson's disease, thereby complicating the diagnostic process for MEDNIK syndrome [3]. Montpetit et al. observed phenotypic changes in MEDNIK syndrome, such as skin changes and severe movement disorders, through knockout of *AP1S1* in zebrafish larvae [2], and they reported that *AP1S1-*knockout embryos often did not survive. To date, 23 mutations in *AP1S1* have been identified, and five mutation sites are reportedly associated with the pathogenesis or possible pathogenesis of MEDNIK syndrome.

The clinical phenotype of MEDNIK syndrome often includes typical symptoms such as developmental delays, intestinal diseases, deafness, peripheral neuropathy, ichthyosis and cutaneous keratosis. We collated data on 16 patients with MEDNIK syndrome, including 14 previously reported patients and the two patients from this study [8] (Table 3). The mutation types of *AP1S1* included insertion, splice-site, frameshift and missense mutations. The variant that we reported was a splice-site mutation. Neurological symptoms in our patients included developmental delays, sensorineural hearing loss, peripheral neuropathy and convulsive seizures [9]. In this study, both patients presented with developmental delays and convulsive seizures. Changes in MRI findings often include brain atrophy, and some patients show highly symmetric signals in the basal ganglia, mainly involving the caudate nucleus and putamen [10]. *AP1S1* mutations can cause peroxidase dysfunction, leading to intestinal epithelial dysfunction [11] and the loss of intestinal ions and macromolecules, leading to congenital intractable diarrhoea. The proband's sister had intractable diarrhoea. Electron microscopy revealed shortened and deteriorated microvilli at the tips of scattered intestinal epithelial cells; duodenal tissue sections showed mild villus atrophy and cytoplasmic vacuoles in intestinal epithelial cells [12]. The clinical manifestations of the two siblings were similar, including developmental delays, convulsive seizures, skin and hair changes, high foreheads and sparse teeth; however, they also had different clinical phenotypes. The proband presented with epilepsy and developmental delay at the onset, followed by ADHD. In contrast, his sister primarily experienced intractable diarrhoea and severe pulmonary infection at the onset, which were considered to be related to the heterogeneity or modifier effects of *AP1S1*. The feature of sparse teeth has not been reported previously. Both children exhibited this characteristic, which may represent a previously unnoticed phenotypic manifestation of MEDNIK syndrome. Further case studies and clinical reports are needed to confirm this observation and to expand our understanding of the phenotypic spectrum of the syndrome.

MEDNIK syndrome often presents with abnormal features such as a high forehead, facial features of Down syndrome, a sunken nose bridge, low ear position and skin lesions [10]. Ichthyosis and keratosis are the most common types of skin lesions. Copper deficiency can lead to decreased tyrosinase activity as well as reduced phenindione and melanin production; therefore, patients with MEDNIK syndrome have reduced skin pigmentation, and in severe cases, hair bleaching can occur. In a previous study, *AP1S1*-knockout zebrafish showed significantly reduced skin pigmentation [2]. Here, both children had fair skin; the proband had manifestations of keratosis cutis, with notably yellow and light hair. His younger sister had light and sparse hair, with reduced pigmentation and light colouration in the middle layer of the hair, as examined under a microscope; furthermore, the inner medullary substance was absent or discoloured. Patients with MEDNIK syndrome occasionally had tricyclic and irregular hair shafts [9]. Under a microscope, typical changes in the hair of patients with Menkes syndrome include a curly texture (shaft torsion), nodular brittleness (transverse shaft breakage), feather brittleness (longitudinal shaft division) and bead-like changes [13]. In cases with similar clinical symptoms and hypoceruloplasminaemia, a preliminary differential diagnosis of MEDNIK syndrome and Menkes syndrome may be made based on microscopic hair changes. However, to determine whether this is a disease-specific change, examination of further samples is necessary.

MEDNIK syndrome has a poor prognosis and a high mortality rate. Of the 14 patients previously reported, eight have died, resulting in a mortality rate of 57% [11]. Currently, no clear treatment options are available for this condition. Zinc acetate has been reported to alleviate copper overload and cholestasis, considerably ameliorating the clinical manifestations and biochemical abnormalities associated with copper overload metabolism [14]. As zinc acetate is not available in China, we attempted to treat the proband's sister (Case 2) with lysine hydrochloride and zinc gluconate granules. After 2 months of treatment, the biochemical indicators of the child did not improve, but the symptoms of intractable diarrhoea and pulmonary hypoxia were relieved. In the absence of zinc acetate, lysine hydrochloride and zinc gluconate granules and other zinc agents may be beneficial to patients. The polarity and bile duct–formation ability of hepatocyte-like cells differentiated from pluripotent stem cells are a focus of research on the treatment of diseases such as MEDNIK syndrome [15].

## 5. Conclusion

In summary, we report two siblings with MEDNIK syndrome in whom a homozygous mutation was found in the c.430-1G>A splice site of *AP1S1*. This mutation leads to the occurrence of splice-3 (i.e., the 3⁣′-end splicing mutation), indicating its possible effect on mRNA production and protein function [16]. To validate this hypothesis, we conducted in vitro mRNA splicing experiments. The experimental results showed that in the mRNA sequence transcribed by the mutant plasmid, Exon 5 had a partial 1-bp deletion, which led to a frameshift mutation in the protein. This protein was annotated as p.Glu144ArgfsTer83. Thus, this locus is a pathogenic variant and represents a previously unreported mutation, enriching the variant spectrum of *AP1S1*, which exhibits genetic heterogeneity. Sparse teeth might be a novel phenotype of MEDNIK syndrome. Examining the microscopic features of hair might become a rapid and convenient method for the differential diagnosis of MEDNIK syndrome and Menkes syndrome. MEDNIK has a high mortality rate; therefore, early and accurate diagnosis is crucial for guiding clinical treatment and providing prenatal diagnosis consultation.

## Figures and Tables

**Table 1:** In silico splicing prediction scores for the c.430-1G>A variant in *AP1S1.*

**Prediction tool**	**Score**	**Threshold**	**Predicted effect on splicing**
dbscSNV AdaBoost (AdaScore)	0.999	> 0.6	Affects splicing (deleterious)
dbscSNV random forest (RFScore)	0.938	> 0.6	Affects splicing (deleterious)
Human Splicing Finder (HSF)	Disruption of canonical acceptor site	—	Affects canonical 3⁣′ splice site

**Table 2:** Behavioural and developmental assessments of the siblings during follow-up visits.

**Patient**	**Assessment scale**	**Item**	**Result**
Proband (9 years and 10 months)	Wechsler Intelligence Test for School-Age Children (WISC-R)	Verbal IQ	< 40
		Operational IQ	< 40
		Total meter points	11 points
		Total IQ < 40	11 points
	Infant-Middle School Student Social Life Ability Scale (S-M)	41 points	Severe developmental delays
	Diagnostic Scale for Attention Deficit Hyperactivity Disorder (DSM-V)	Positive attention deficit symptoms	4 items
		Positive symptoms of hyperactivity and impulsivity	1 item
	Attention Deficit Hyperactivity Behaviour Scale (ADHD)	Total number of attention deficit symptoms	8/9
		Total number of hyperactive impulsive symptoms	6/9
		Total number of mixed symptoms	14/18
		Learn the total number of symptoms	5/8
	Weiss Deficit Assessment Scale-Parent Version (WFIRS-P)	Family	Damage
		Study and school	Damage
		Life skills	Damage
		Self-management	Damage
		Social activities	Damage
		Adventure activities	Normal
	Parent and Teacher Rating Scale for Attention Deficit Hyperactivity Disorder (SNAP-IV)	Attention deficit 23 points	Severe abnormalities
		Hyperactive/impulsive 17 points	Moderate abnormality
		Oppositional defiant 8 points	Normal

Proband's sister (4 years and 8 months)	Checklist of neuropsychological development in children aged 0–6 years	Great exercise	22.5 months (DQ43)
		Fine movements	18 months (DQ35)
		Adaptability	22.5 months (DQ43)
		Language	15 (DQ29)
		Social behaviour	18 months (DQ35)
		Full scale	19 March (DQ37)
		SM	23 (moderately abnormal)
	Schedule of early language development	Speech and language expression	11 points (equivalent to 15 months)
		Auditory perception and comprehension	13 points (equivalent to 17–18 months)
		Understanding and expression related to vision	12 points (equivalent to 12–13 months)
		36 on the full scale	36 points (equivalent to 15–16 months)
	Psychological test report	ABC	Negative
		CARS	Negative
		DSM-V	Negative

**Table 3:** Clinical information of patients reported previously.

**Patient number**	**1**	**2**	**3**	**4**	**5**	**6**	**7**	**8**	**9**	**10**	**11**	**12**	**13**	**14**	**15**	**16**
Authors	Martinelli et al.	Martinelli et al.	Martinelli et al.	Martinelli et al.	Martinelli et al.	Martinelli et al.	Martinelli et al.	Incecik et al.	Klee et al.	Klee et al.	Klee et al.	Jiajie et al.	Rackova et al.	Rackova et al.	Us	Us
Nation	Canada	Canada	Canada	Canada	Canada	Canada	Italy	Türkiye	Australia	Australia	Australia	America	Czech	Czech	China	China
Sex	F	F	F	M	F	F	nd	F	F	F	F	F	F	M	M	F
Age	8 years	27 years	28 years	19 years	1.5 years	2 months	nd	10 years	1 month	21 days	3 months	6 months	18 weeks	5.5 years	5 years	3 months
Mortality	−	+	−	−	+	+	−	−	+	+	+	−	+	+	−	−
Genotype	356_365insG	IVS2-2A>G	IVS2-2A>G	IVS2-2A>G	IVS2-2A>G	IVS2-2A>G	nd	p.D122Gfs∗18(c.356_357insG)	c.269T>C	c.269T>C	c.346G>A	C.186T>G	c.269T>C	c.269T>C	c.430-1G>A	c.430-1G>A
Mental retardation	+	+	+	+	+	+	nd	+	nd	nd	nd	+	nd	+	+	+
Intestinal disease	+	+	+	+	+	+	+	+	+	+	+	+	+	+	nd	+
Deafness	+	+	+	+	+	−	+	+	nd	nd	nd	+	−	+	−	−
Neuropathy	−	+	+	+	nd	−	nd	−	nd	nd	nd	+	−	+	+	+
Abnormalities of skin and hair	+	+	+	+	+	+	+	+	nd	nd	nd	nd	+	+	+	+
Liver function injury	+	+	+	+	+	+	nd	+	nd	nd	+	+	+	+	+	+
High ALP	+	+	+	−	nd	nd	nd	+	nd	nd	nd	nd	nd	nd	+	+
Low serum copper	+	+	+	+	nd	nd	+	+	nd	nd	nd	+	nd	nd	+	−
Low ceruloplasmin	+	+	+	+	nd	nd	+	+	nd	nd	nd	+	+	+	+	+
High VLCFA	+	+	+	+	+	nd	nd	+	nd	nd	nd	nd	nd	−	−	−
MRI cerebral atrophy	+	+	+	+	+	nd	nd	+	nd	nd	nd	nd	nd	nd	−	−
MRI basal ganglia abnormalities	+	+	−	+	−	nd	nd	−	nd	nd	nd	nd	nd	nd	−	−

Abbreviation: nd, not determined.

## Data Availability

All data generated or analysed during the study are included in this manuscript. Further enquiries can be directed to the corresponding authors.

## References

[1] Saba T. G., Montpetit A., Verner A. (2005). An Atypical Form of Erythrokeratodermia Variabilis Maps to Chromosome 7q22. *Human Genetics*.

[2] Montpetit A., Côté S., Brustein E. (2008). Disruption of *AP1S1*, Causing a Novel Neurocutaneous Syndrome, Perturbs Development of the Skin and Spinal Cord. *PLoS Genetics*.

[3] Alsaif H. S., Al-Owain M., Barrios-Llerena M. E. (2019). Homozygous Loss-of-Function Mutations in AP1B1, Encoding Beta-1 Subunit of Adaptor-Related Protein Complex 1, Cause MEDNIK-Like Syndrome. *American Journal of Human Genetics*.

[4] Hu Frisk J. M., Kjellén L., Kaler S. G., Pejler G., Öhrvik H. (2017). Copper Regulates Maturation and Expression of an MITF:Tryptase Axis in Mast Cells. *Journal of Immunology*.

[5] Culotta V. C., Gitlin J. D., Valle D. L. (2019). Disorders of Copper Transport. *The Online Metabolic and Molecular Bases of Inherited Disease*.

[6] Ferreira C. R., Gahl W. A. (2017). Disorders of Metal Metabolism. *Translational Science of Rare Diseases*.

[7] Bonifacino J. S. (2014). Adaptor Proteins Involved in Polarized Sorting. *Journal of Cell Biology*.

[8] Rackova M., Mattera R., Svaton M. (2024). Revising Pathogenesis of AP1S1-Related MEDNIK Syndrome: A Missense Variant in the *AP1S1* Gene as a Causal Genetic Lesion. *Journal of Molecular Medicine*.

[9] Martinelli D., Travaglini L., Drouin C. A. (2013). MEDNIK Syndrome: A Novel Defect of Copper Metabolism Treatable by Zinc Acetate Therapy. *Brain*.

[10] Incecik F., Bisgin A., Yılmaz M. (2018). MEDNIK Syndrome With a Frame Shift Causing Mutation in *AP1S1* Gene and Literature Review of the Clinical Features. *Metabolic Brain Disease*.

[11] Klee K. M. C., Janecke A. R., Civan H. A. (2020). AP1S1 Missense Mutations Cause a Congenital Enteropathy via an Epithelial Barrier Defect. *Human Genetics*.

[12] Lu J. G., Namjoshi S. S., Niehaus A. D. (2023). Clinicopathologic Features of IDEDNIK (MEDNIK) Syndrome in a Term Infant: Histopathologic Features of the Gastrointestinal Tract and Report of a Novel AP1S1 Variant. *Pediatric and Developmental Pathology*.

[13] Rangarh P., Kohli N. (2018). Neuroimaging Findings in Menkes Disease: A Rare Neurodegenerative Disorder. *BML Case Reports*.

[14] Martinelli D., Dionisi-Vici C. (2014). AP1S1 Defect Causing MEDNIK Syndrome: A New Adaptinopathy Associated With Defective Copper Metabolism. *Annals of the New York Academy of Sciences*.

[15] Overeem A. W., Klappe K., Parisi S. (2019). Pluripotent Stem Cell-Derived Bile Canaliculi-Forming Hepatocytes to Study Genetic Liver Diseases Involving Hepatocyte Polarity. *Journal of Hepatology*.

[16] O'Neill M. J., Yang T., Laudeman J. (2024). ParSE-Seq: A Calibrated Multiplexed Assay to Facilitate the Clinical Classification of Putative Splice-Altering Variants. *Nature Communications*.

